# In vitro antimicrobial activity of *Naja* cardiotoxin peptide-3 against clinical isolates from canine otitis

**DOI:** 10.1007/s11259-026-11284-3

**Published:** 2026-05-23

**Authors:** Costanza Spadini, Nicolò Mezzasalma, Alessandro Cupola, Simone Taddei, Clotilde Silvia Cabassi

**Affiliations:** Department of Veterinary Science, University of Parma, Strada del Taglio 10, Parma, 43126 Italy

**Keywords:** Antimicrobial peptides, Antimicrobial resistance, *M. pachydermatis*, *Naja atra*, Cardiotoxin, NCP-3.

## Abstract

Canine otitis externa is a common multifactorial condition frequently complicated by bacterial and fungal infections. The increasing prevalence of antimicrobial resistance has reduced the effectiveness of conventional therapies, underscoring the need for alternative treatment strategies. Antimicrobial peptides represent a promising option due to their broad-spectrum activity and low propensity to induce resistance. Among these, the *Naja* Cardiotoxin Peptide-3 (NCP-3), derived from cardiotoxin 1 of the Chinese cobra (*Naja atra* subsp. *atra*), has demonstrated potent bactericidal and fungicidal activity with minimal cytotoxicity. In this study, the in vitro antimicrobial activity of NCP-3 was evaluated against clinical isolates obtained from dogs with otitis externa, including multidrug-resistant bacteria and *Malassezia pachydermatis*. NCP-3 was effective against all reference tested strains except *Proteus mirabilis*. Among field strains, the highest bactericidal activity was observed on *Staphylococcus pseudintermedius* (MBC₅₀=3.1 µg/mL; MBC₉₀=6.3 µg/mL), whereas higher concentrations were required to inhibit Pseudomonadaceae (MBC₅₀=12.5 µg/mL). Neither MBC₅₀ nor MBC₉₀ were achieved for Enterobacteriaceae or *M. pachydermatis*. Time-kill assays demonstrated rapid bactericidal activity of NCP-3 against both reference and clinical Gram-positive strains, with complete inhibition achieved within five minutes. In contrast, Gram-negative reference strains required longer exposure times, with a partial inhibition only at 120 min. Overall, these findings indicate that NCP-3 exhibits stronger antimicrobial activity against Gram-positive bacteria, including resistant strains, than on Gram-negative bacteria and *M. pachydermatis* field strains. NCP-3 may therefore represent a promising candidate as therapeutic agent for canine otitis externa caused by Gram-positive pathogens, supporting further investigation into its potential applications in veterinary medicine.

**Supplementary Information:**

The online version contains supplementary material available at 10.1007/s11259-026-11284-3.

## Introduction

Canine otitis externa is one of the most common diseases in companion animals, characterized by a multifactorial etiology and often triggered or exacerbated by bacterial and yeast infections (Saraiva et al. 2025). Both Gram-positive bacteria such as *Staphylococcus* spp., *Streptococcus* spp., and *Enterococcus* spp. and Gram-negative bacteria, including *Pseudomonas* spp., *Proteus* spp., *Escherichia coli*, and other members of the *Enterobacteriaceae* family, are commonly implicated in canine otitis. In addition, the zoonotic potential of certain *Staphylococcus* species isolated from canine otitis externa and other dermatological conditions is well documented (Dégi et al. 2013; Popa et al. 2025). Current guidelines for the management of ear infections in dogs recommend topical therapy for both otitis externa and otitis media (Guardabassi et al. 2008). The concentration of antimicrobials in topical formulations depends on several factors, including the antimicrobial spectrum of the active compound, the causative microorganism, and the integrity of the tympanic membrane (Guardabassi et al. 2008). In recent years, increasing concern has focused on the rising prevalence of antimicrobial-resistant bacteria, particularly *Staphylococcus* spp. and *Pseudomonas* spp., in cases of canine otitis externa (Bourély et al. 2019; Ghibaudo et al. 2016) which has significantly reduced the effectiveness of conventional antimicrobial therapies. This phenomenon is largely attributed to inappropriate or prolonged antibiotic use, which promotes the development of bacterial resistance mechanisms, such as efflux pumps in *Pseudomonas aeruginosa* (Morita et al. 2013). Yeasts, including *Malassezia* spp. and *Candida* spp., also play a crucial role in the pathogenesis of otitis externa (Guillot and Bond 2020; Saridomichelakis et al. 2007; Weese 2013). Of particular concern is the increasing number of *Malassezia* spp. strains showing resistance to commonly used azole antifungals (Kano et al. 2019), potentially compromising the efficacy of standard antifungal treatments.

The growing issue of antimicrobial resistance has prompted increasing interest in alternative therapeutic strategies for the management of infections caused by resistant bacteria and fungi (Callaway et al. 2021; Mezzasalma et al. 2025; Spadini et al. 2025). Among these, antimicrobial peptides (AMPs) have emerged as promising candidates due to their broad-spectrum activity against Gram-positive and Gram-negative bacteria, as well as viruses, fungi, and parasites (Sala et al. 2018). AMPs may serve as alternatives to conventional antibiotics or act synergistically with them, owing to their rapid mode of action and relatively low propensity to induce resistance (Romani et al. 2013; Yeaman and Yount 2003).

Cationic antimicrobial peptides have attracted considerable attention (Hiemstra and Zaat 2013). These peptides initially interact with negatively charged components on microbial surfaces through electrostatic interactions (Brogden 2005). In Gram-negative bacteria, they bind to anionic phospholipids and phosphate groups of lipopolysaccharides, whereas in Gram-positive bacteria, they primarily interact with teichoic acids within the peptidoglycan layer. This initial interaction ultimately leads to pore formation, membrane disruption, and microbial cell death (Sato and Feix 2006; Zasloff 2019). In addition, certain AMPs exhibit antifungal activity by inhibiting the synthesis of galactosylceramide, a key component of fungal cell membranes, thereby impairing membrane integrity and inhibiting fungal growth (Thevissen et al. 2004).

Antimicrobial peptides are synthesized in various tissues of vertebrates and invertebrates as part of the innate immune response, such as defensins, and have also been identified in animal venoms, where they function as toxins (Dubovskii et al. 2015; Sala et al. 2018). When isolated from venom, AMPs often retain antimicrobial activity while exhibiting reduced cytotoxicity (Dubovskii et al. 2015). A novel family of AMPs has been developed based on cardiotoxin-1 (CTX-1) from the Chinese cobra (*Naja atra* subsp. *atra*) (Sala et al. 2018). These peptides demonstrated potent antibacterial and antifungal activity against a broad panel of bacterial and fungal reference strains, with minimal cytotoxic and hemolytic effects on eukaryotic cells. Among them, Naja cardiotoxin peptide-3 (NCP-3) exhibited the highest bactericidal and fungicidal activity, as well as rapid onset of action. Furthermore, NCP-3 has shown activity against mycobacteria and the enveloped virus Bovine herpesvirus 1 (BoHV-1) (Sala et al. 2018).

The aim of the present study was to evaluate the in vitro antibacterial and antifungal activity of NCP-3 against bacterial and yeast isolates obtained from dogs with otitis externa, including strains with known antimicrobial resistance profiles.

## Materials and methods

### NCP-3

The design of Naja cardiotoxin peptide-3 (NCP-3), based on the amino acid sequence of S-type cardiotoxin 1 (CTX-1) from the Chinese cobra (*Naja atra* subsp. *atra*), has been previously described, together with the physicochemical characteristics of the peptide (Sala et al. 2018). NCP-3 was synthesized by Selleck-Chem (Houston, TX, USA) and stored as a lyophilized powder at − 20 °C until use.

### Bacterial and fungal strains and identification

All clinical and reference strains included in the study are listed in Supplementary Table S1.

Clinical isolates were obtained from the biobank of the Animal Infectious Disease Laboratory, University of Parma. A total of 53 bacterial clinical isolates were analyzed, including *Burkholderia cepacia* (n = 3), *Escherichia coli* (n = 9), *Enterobacter cloacae (2)*,* Proteus mirabilis* (*n* = 4), *Pseudomonas aeruginosa* (n = 18), *Staphylococcus pseudintermedius (n = 17)*. Fourteen clinical yeast isolates of *Malassezia pachydermatis* were also included.

The following reference strains were tested: *E. coli* ATCC 25,922; *P. aeruginosa* ATCC 27,853, *P. mirabilis* ATCC 14,153, *S. aureus* ATCC 25,923, methicillin-resistant *Staphylococcus aureus* (MRSA) ATCC 43,300, *S. pseudintermedius* ATCC 21,284, and *M. pachydermatis* DSM 6172.

Clinical isolates were obtained from ear canal swabs collected from dogs diagnosed with otitis externa and presented at the Veterinary Teaching Hospital, Department of Veterinary Science, University of Parma. Samples were collected using sterile swabs with Amies transport medium and immediately plated onto Columbia blood agar, MacConkey agar, and Sabouraud dextrose agar (Difco, Sparks, USA). Plates were incubated aerobically at 37 °C for 24–48 h.

Bacterial isolates were identified based on colony morphology, Gram staining, cellular morphology, catalase and oxidase reactions, and API biochemical test system (bioMérieux, France) (Quinn P. J, 1994). Species-level identification of *Staphylococcus* spp. was confirmed using MALDI-TOF MS (Bruker Biotyper, Bruker Daltonics GmbH, Bremen, Germany). Yeast identification was initially based on colony morphology and methylene blue staining (Quinn P. J, 1994). Isolates showing typical morphology of *M. pachydermatis* were subcultured on CHROMagar™ *Malassezia* medium (CHROMagar, Saint-Denis, France). Colonies with a mucoid pink-to-purple appearance were replated on Sabouraud dextrose agar and incubated at 37 °C for 48 h.

Molecular identification of *M. pachydermatis* was performed by nested PCR targeting the internal transcribed spacer 1 (ITS1) region of the rRNA gene, as previously described (Spadini et al. 2025). Briefly, genomic DNA was extracted using the MasterPure Yeast DNA Purification Kit (Epicentre Technologies), suspended in Tris–EDTA buffer, and stored at − 20 °C until analysis.

### Antimicrobial susceptibility testing (AST) of bacteria and yeast strains

Antimicrobial susceptibility testing of bacterial isolates was performed using the Kirby–Bauer disk diffusion method, except for polymyxin B, which was tested by broth microdilution to determine the minimum inhibitory concentration (MIC). The antimicrobial agents tested reflected those most commonly used for the clinical management of canine otitis at the Veterinary Teaching Hospital and included amoxicillin/clavulanic acid (20/10 µg), cephalexin (30 µg); cefadroxil (30 µg); cefovecin (30 µg); ciprofloxacin (5 µg); clindamycin (2 µg); doxycycline (30 µg); enrofloxacin (5 µg), florfenicol (30 µg), gentamicin (10 µg), marbofloxacin (5 µg), oxytetracycline (30 µg), oxacillin (30 µg), pradofloxacin (5 µg) and trimethoprim + sulfamethoxazole (25 µg).

Polymyxin B MICs were determined using concentrations ranging from 256 to 0.5 µg/mL. Bacterial inocula were prepared according to CLSI guidelines (CLSI, 2024). Results were interpreted following CLSI standards (VET01S and M100). When veterinary-specific breakpoints were unavailable, human clinical breakpoints were applied (Clinical and Laboratory Standards Institute 2021; Clinical Laboratory Standards Institute, 2023) Multidrug-resistant (MDR) status was assigned according to previously published criteria (Magiorakos et al. 2012) Susceptibility of *M. pachydermatis* isolates to azole antifungals was evaluated by MIC determination using ketoconazole (KTZ), miconazole (MCZ), and fluconazole (FCZ) (Sigma-Aldrich). *M. pachydermatis* DSM 6172 was used as a quality control strain.

### Fungal inoculum preparation and MIC determination

Fungal inocula were prepared following CLSI reference methods for yeast susceptibility testing, with minor modifications (CLSI, 2017; Spadini et al. 2025). Yeast isolates stored at − 80 °C were cultured in modified RPMI 1640 broth (Gibco, Life Technologies Ltd), supplemented as described in the literature (Rojas et al. 2014), and incubated at 37 °C for 48 h.

Four to five colonies (~ 1 mm diameter) were suspended in 10 mM phosphate buffer (PB; pH 7.0) and adjusted to a 0.5 McFarland standard, corresponding to approximately 1 × 10⁶–5 × 10⁶ CFU/mL. Growth and sterility controls, with and without 1% DMSO, were included.

KTZ, MCZ, and FCZ were dissolved in DMSO (25.6 mg/mL) and tested at final concentration ranges of 0.008–32 µg/mL for KTZ and 0.125–256 µg/mL for MCZ and FCZ. After 48 h of incubation at 37 °C, 10 µL of 0.02% resazurin in PBS (pH 7.0) was added to each well. Plates were incubated for an additional 24 h before MIC determination (Palomino et al. 2002). A color change from blue to pink indicated fungal growth. MIC values were defined as the lowest drug concentration preventing color change.

Each experiment was performed in triplicate with three technical replicates. MIC values were expressed as mean ± standard deviation. As CLSI breakpoints for *M. pachydermatis* azole susceptibility are not available, MIC₅₀ and MIC₉₀ values were calculated.

### Bacterial inoculum preparation and MIC determination

Bacterial inocula were prepared according to CLSI guidelines (Clinical and Laboratory Standards Institute 2021). Five colonies from fresh cultures were inoculated into Mueller–Hinton broth (MHB) and incubated at 37 °C in aerobiosis for 24 h. After incubation, cultures were centrifuged at 2,000 rpm for 20 min at 4 °C, and pellets were resuspended in 10 mM PB (pH 7.0). Suspensions were adjusted spectrophotometrically (600 nm) to an optical density of 0.08–0.13, corresponding to approximately 1 × 10⁸ CFU/mL, and subsequently diluted 1:100 to obtain a final concentration of 5 × 10⁵ CFU/mL.

### Minimum bactericidal concentration (MBC) assay

The minimum bactericidal concentration of NCP-3 was determined using a broth microdilution method following CLSI guidelines (Clinical and Laboratory Standards Institute 2021). Serial dilutions of NCP-3 were prepared in PB (10 mM, pH 7.0) at a concentration range of 0.8–12.5 µg/mL, based on MBC values obtained in a previous study on reference bacterial strains (Sala et al. 2018), and selected to focus on clinically relevant concentrations and those potentially achievable in a topical formulation for the treatment of canine otitis externa. The peptide dilutions were incubated with bacterial or yeast suspensions in 96-well U-bottom microtiter plates at 37 °C for 2 h.

Following incubation, 20 µL from each well was plated onto Mueller–Hinton agar for bacteria or Sabouraud dextrose agar for yeasts and incubated at 37 °C for 24 h. Colonies were counted, and inhibition percentages were calculated using the following formula:

$$\:\mathrm{I}\mathrm{n}\mathrm{h}\mathrm{i}\mathrm{b}\mathrm{i}\mathrm{t}\mathrm{i}\mathrm{o}\mathrm{n}\:\left(\mathrm{\%}\right)=\frac{{\stackrel{-}{\mathrm{C}\mathrm{F}\mathrm{U}}}_{\mathrm{G}\mathrm{C}}-{\stackrel{-}{\mathrm{C}\mathrm{F}\mathrm{U}}}_{\mathrm{T}\mathrm{C}}}{{\stackrel{-}{\mathrm{C}\mathrm{F}\mathrm{U}}}_{\mathrm{G}\mathrm{C}}}*100$$

where GC represents growth control and TC the tested concentration. Each assay was performed in triplicate, with growth and sterility controls included. The MBC of NCP-3 was defined as the minimum concentration required to achieve ≥ 99.9% killing of the initial inoculum. Data are reported as mean ± standard deviation (SD) based on biological replicates for each strain.

For clinical isolates, summary measures were used, including MBC₅₀ and MBC₉₀, defined as the minimum bactericidal concentrations required to kill 50% and 90% of the isolates, respectively.

### Time kill assay (TKA)

Time–kill assays were performed to evaluate the antimicrobial activity of NCP-3 over time using a broth microdilution method at 2× MBC, as previously described (Romani et al. 2013). For clinical isolates for which the MBC was not achieved within the tested concentration range (≤ 12.5 µg/mL), the assay was conducted at 2× the highest tested concentration (i.e., 25 µg/mL).

Aliquots (20 µL) were collected at 5, 10, 15, 30, 60, and 120 min and plated onto Mueller–Hinton agar or Sabouraud dextrose agar. After 24 h of incubation at 37 °C, CFU counts for bacteria and cell counts for yeasts were determined. Inhibition percentages were calculated as described for the MBC assay. Growth and sterility controls were included in all experiments.

### Statistical analysis

Data were analyzed using SPSS version 29.1 (IBM Corp., Armonk, NY, USA). Normality of MBC values was assessed using the Kolmogorov–Smirnov test. Differences among groups were evaluated using the Kruskal–Wallis test, followed by post hoc pairwise comparisons with Bonferroni correction. For clinical strains for which the MBC was not reached at the highest tested concentration (12.5 µg/mL), the next step in the two-fold dilution series (25 µg/mL) was assigned for statistical analyses, including the Kruskal-Wallis test.

## Results

### Antimicrobial susceptibility testing of bacterial and yeast field strains

The results of antimicrobial susceptibility testing for bacterial and yeast field strains are reported in the Supplementary Materials (Tables S2 and S3). Overall, on marbofloxacin the field strains considered showed the lowest level of resistance (39.62%). Regarding Pseudomonadaceae family, they exhibited a high level of resistance to most of the tested antimicrobial agents; however, three *P. aeruginosa* and two *B. cepacia* isolates were resistant to the entire antimicrobial panel. Except for one isolate (MIC = 1 µg/mL), all Pseudomonadaceae field strains showed MIC values ≤ 0.5 µg/mL for polymyxin B.

Among Enterobacteriaceae isolates, approximately 80% were classified as multidrug-resistant, showing resistance to multiple antimicrobial classes, including aminoglycosides, β-lactams, penicillins combined with β-lactamase inhibitors, and quinolones. With respect to polymyxin B, two *P. mirabilis* isolates exhibited high-level resistance (MIC > 256 µg/mL), whereas all remaining Enterobacteriaceae strains showed MIC values ≤ 0.5 µg/mL.

Nearly all *S. pseudintermedius* isolates were resistant to oxacillin (16/17; 94.12%), as assessed by oxacillin-based phenotypic testing according to current EUCAST/CLSI recommendations, confirming the presence of methicillin-resistant strains (CLSI, 2024). Overall, 14/17 isolates (82.35%) were classified as multidrug-resistant (MDR), showing resistance to three or more antimicrobial classes (Magiorakos et al. 2012). In addition, most strains displayed resistance to several other antimicrobial classes, including lincosamides, tetracyclines, quinolones, and folate pathway inhibitors.

Among *M. pachydermatis* field strains, 8 of 14 isolates were classified as susceptible to ketoconazole (MIC₅₀ = 0.016 µg/mL; MIC₉₀ = 8.0 µg/mL), one isolate was resistant (MIC = 16 µg/mL), and five exhibited intermediate susceptibility. For miconazole, 9 of 14 isolates were susceptible (MIC₅₀ = 32.0 µg/mL; MIC₉₀ = 64.0 µg/mL), one isolate was resistant (MIC = 128 µg/mL), and four were classified as intermediate. With respect to fluconazole, 10 of 14 isolates were susceptible (MIC₅₀ = 32.0 µg/mL; MIC₉₀ = 64.0 µg/mL), one isolate was resistant (MIC = 128 µg/mL), and three exhibited intermediate susceptibility.

### Minimum bactericidal concentration (MBC) assay

#### Reference strains

The MBC values obtained for bacterial and yeast reference strains are summarized in Table 1. At the tested concentrations, an MBC was not achieved only for *P. mirabilis*. The lowest MBC value was observed for *S. pseudintermedius* (0.8 µg/mL). Identical MBC values (6.3 µg/mL) were recorded for *P. aeruginosa*, *E. coli*, *S. aureus*, and *M. pachydermatis*. The highest MBC value was observed for MRSA (12.5 µg/mL).

**Table 1:** Minimum Bactericidal Concentration (MBC) of Naja Cardiotoxin Peptide-3 (NCP-3) against reference bacterial and yeast strains. MBC values represent the minimum concentration required to achieve ≥ 99.9% killing of the initial inoculum. Data are expressed as mean ± standard deviation (SD) based on biological replicates for each strain

References strains	MBC (µg/mL)	SD
*P. aeruginosa* ATCC 27,853	6.3	0
*E. coli* ATCC 25,922	6.3	0
*S. aureus* ATCC 25,923	6.3	0
MRSA ATCC 43,300	12.5	0
*P. mirabilis* ATCC 14,153	> 12.5	0
*S. pseudintermedius* ATCC 21,284	0.8	0
*M. pachydermatis* DSMZ 6172	6.3	0

#### Clinical isolates

Table 2 reports the MBC results for clinical isolates, grouped by bacterial family, and expressed as MBC₅₀ and MBC₉₀ values. For Enterobacteriaceae and *M. pachydermatis* field isolates MBC₅₀ and MBC₉₀ was not achieved, due to a high proportion of clinical isolates whose MBC exceeded the higher tested concentration. In contrast, MBC₅₀ and MBC₉₀ values of 3.1 and 6.3 µg/mL, respectively, were observed for *S. pseudintermedius* isolates. For Pseudomonadaceae, only an MBC₅₀ value (12.5 µg/mL) was obtained.

**Table 2:** Minimum Bactericidal Concentration of *Naja* Cardiotoxin Peptide-3 (NCP-3) required to kill 50% (MBC_50_) and 90% (MBC_90_) of the field strains

Field strains	µg/ml	
	MBC_50_	MBC_90_
Enterobacteriaceae (*n* = 15)	> 12.5	> 12.5
Pseudomonadaceae (*n* = 21)	12.5	> 12.5
*S. pseudintermedius* (*n* = 17)	3.1	6.3
*M. pachydermatis* (*n* = 14)	> 12.5	> 12.5

**Fig. 1:**
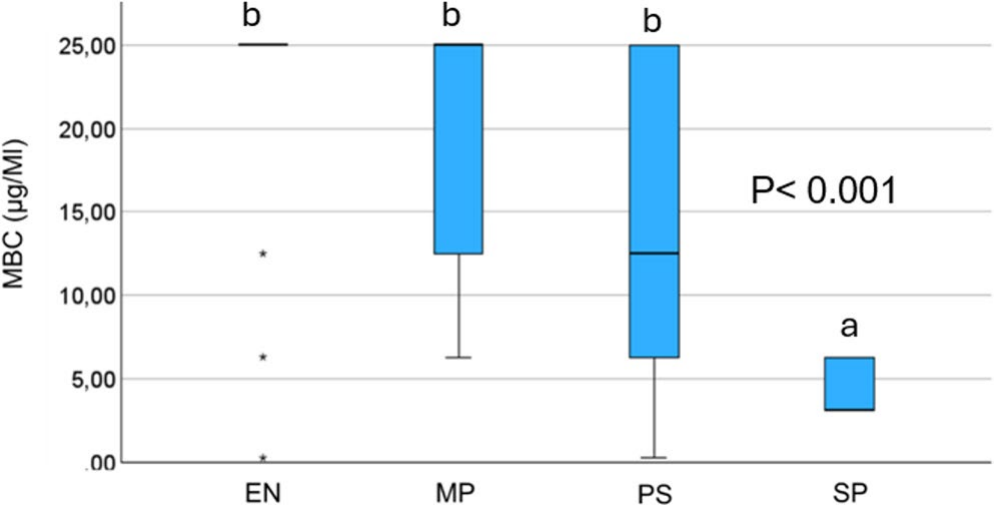
Comparison of Minimum Bactericidal Concentration (MBC) values of Naja Cardiotoxin Peptide-3 (NCP-3) among clinical isolates from different microorganism families, analyzed using the Kruskal–Wallis test. EN= Enterobacteriaceae (n = 15); MP = *M. pachydermatis* (n = 14); PS = Pseudomonadaceae (n = 21); SP = *S. pseudintermedius* (n = 17). Values exceeding 12.5 µg/mL were assigned a value of 25 µg/mL for visualization and statistical analysis. NCP-3 exhibited significantly lower MBC values against *S. pseudintermedius* compared to other groups (p < 0.01), indicating greater antimicrobial activity against Gram-positive isolates, whereas no significant differences were observed among Enterobacteriaceae, Pseudomonadaceae, and *M. pachydermatis*

A statistically significant difference in MBC values among the tested groups was observed (*P* < 0.001), with the highest bactericidal activity recorded against *S. pseudintermedius* isolates (Fig. 1).

Different superscript letters (a, b) indicate statistically significant differences between groups; groups sharing the same letter are not significantly different.

### Time kill assay (TKA)

**Fig. 2:**
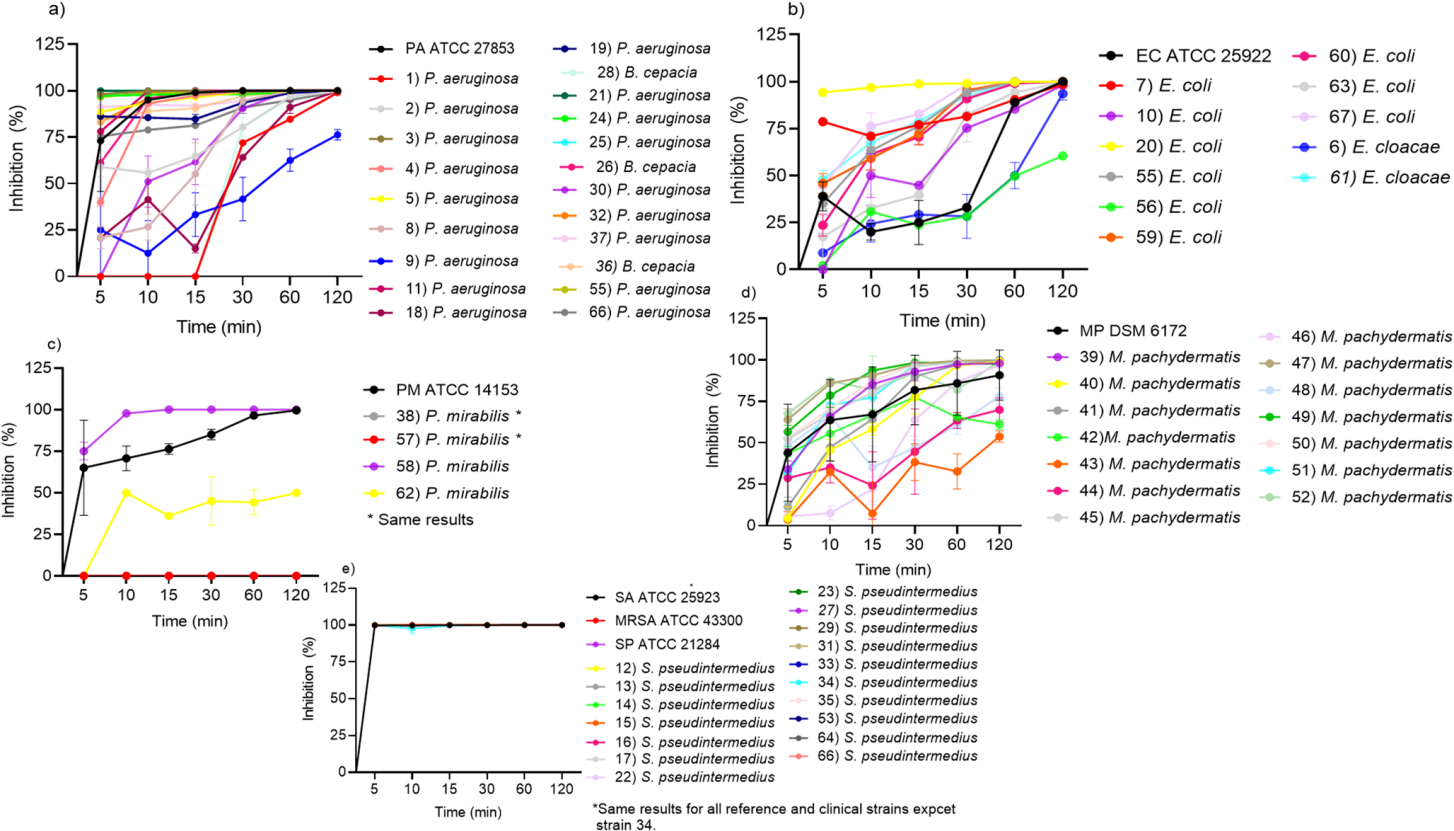
**(a-e):** Time–kill kinetics of Naja Cardiotoxin Peptide-3 (NCP-3) against reference and clinical bacterial and fungal strains. Each panel shows changes in viable counts over time following exposure to NCP-3 at the indicated concentrations. Error bars represent standard deviation (SD) from biological replicates. PA = *P. aeruginosa*; SA = *S. aureus*; MRSA = methicillin-resistant *S. aureus*; EC = *E. coli*; PM = *P. mirabilis*; SP = *S. pseudintermedius*; MP = *M. pachydermatis*. NCP-3 exhibited rapid and pronounced bactericidal activity against Gram-positive strains, with near-complete killing achieved within minutes, whereas Gram-negative bacteria showed slower and incomplete killing. Variable reduction in viability was observed for *M. pachydermatis* under the tested conditions

**Fig. 3:**
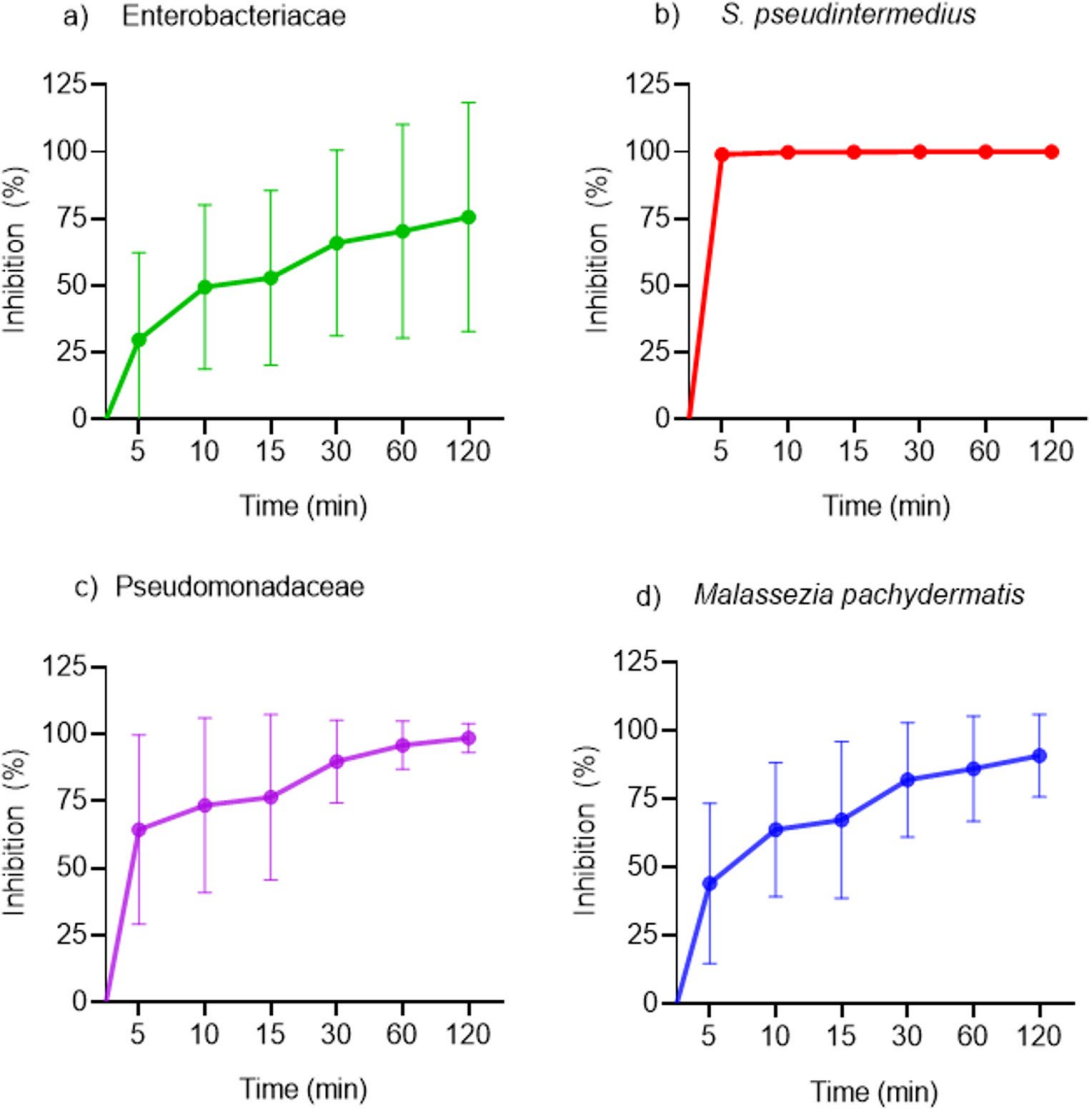
**(a-d):** Time–kill kinetics of Naja Cardiotoxin Peptide-3 (NCP-3) against clinical isolates grouped by taxonomic family. Each panel represents the mean change in viable counts over time following exposure to NCP-3. Error bars indicate standard deviation (SD) across strains within each family and do not account for biological replicates of individual isolates. Enterobacteriaceae (n = 15); *M. pachydermatis* (n = 14); Pseudomonadaceae (n = 21); *S. pseudintermedius* (n = 17). NCP-3 induced rapid and complete killing in *S. pseudintermedius*, whereas a gradual and incomplete reduction in viability was observed for Enterobacteriaceae, Pseudomonadaceae and *M. pachydermatis*

The results of the time–kill assays performed using NCP-3 at 2× MBC are shown in Fig. 2 (a–e) for individual reference and clinical strains, while results for field strains grouped by family are presented in Fig. 3 (a–d).

NCP-3 exhibited rapid and pronounced bactericidal activity against Gram-positive strains, with near-complete killing achieved within minutes, whereas Gram-negative bacteria showed slower and incomplete killing. Variable reduction in viability was observed for *M. pachydermatis* under the tested conditions.

#### Reference strains

Among reference strains, *S. aureus*, MRSA, *S. pseudintermedius*, and *M. pachydermatis* were completely inhibited within 5 min of exposure to NCP-3. At the same time point, inhibition rates of 40%, 65%, and 70% were observed for *E. coli*, *P. mirabilis*, and *P. aeruginosa*, respectively. *P. aeruginosa* showed near-complete inhibition after 15 min of exposure. In contrast, *E. coli* exhibited ≥ 90% of the inhibition only after 60 min, reaching complete inhibition at 120 min. *P. mirabilis* demonstrated a gradual increase in inhibition, ranging from 60% to 80% between 5 and 30 min, with complete inhibition achieved only after 120 min.

#### Clinical isolates

After 5 min of exposure, the highest antimicrobial activity was observed against *S. pseudintermedius* field strains, for which complete inhibition was recorded in all tested isolates. In contrast, isolates belonging to the Pseudomonadaceae, Enterobacteriaceae, and *M. pachydermatis* groups showed marked variability in susceptibility to NCP-3 over time. Within Pseudomonadaceae, inhibition varied considerably during the first 30 min of exposure but reached 96% at 60 min and increased to 98.7% at 120 min. Greater variability was observed among Enterobacteriaceae isolates; therefore, results are presented by bacterial genus in Figs. < link rid="fig1”>3</link>-b and 3-c. *E. coli* and *Enterobacter cloacae* isolates showed partial inhibition up to 60 min. At this time point, all strains except two (one *E. coli* and one *E. cloacae*) exhibited at least 96% inhibition, while after 120 min all but one isolate reached 99% inhibition. *Proteus mirabilis* field strains displayed low susceptibility to NCP-3, with two of four isolates showing no inhibition throughout the assay. One isolate achieved complete inhibition after 15 min, whereas the remaining isolate reached only 50% inhibition at 120 min. Field isolates of *M. pachydermatis* (Fig. 3d) also exhibited high variability in susceptibility at all evaluated time points. Only two of fourteen isolates achieved ≥ 99% inhibition after 60 min, while six isolates reached this level of inhibition after 120 min. When evaluating time–kill assays grouped by bacterial family (Fig.3 a-d), only the S. pseudintermedius group showed low variability across the examined time points, with minimal or no standard deviation among isolates, and complete inhibition was achieved within 5 min. In contrast, Enterobacteriaceae, Pseudomonadaceae, and *M. pachydermatis* clinical isolates exhibited a time-dependent increase in inhibition, although accompanied by high variability among isolates, as reflected by the larger standard deviations.

## Discussion

In recent years, increasing attention has been directed toward the development of alternative antimicrobial agents for use in both veterinary and human medicine. Among these, antimicrobial peptides (AMPs) represent a promising option due to their structural properties and membrane-active mechanisms against pathogenic microorganisms (Oliveira Júnior et al. 2025). The present study builds upon previous research on peptides derived from cardiotoxin-1 (CTX-1) of the Chinese cobra (*Naja atra* subsp. *atra*). Among these peptides, Naja cardiotoxin peptide-3 (NCP-3) has shown particularly promising antimicrobial activity (Sala et al. 2018). Compared with previous findings (Sala et al. 2018), NCP-3 showed slightly lower MBC values against reference strains of *E. coli* (from 12.5 to 6.3 µg/mL) and *P. aeruginosa* (from 12.5 to 6.3 µg/mL), whereas higher values were observed for MRSA (from 6.3 to 12.5 µg/mL) and *S. aureus* (from 1.6 to 6.3 µg/mL). Similar results were obtained for *P. mirabilis* (MBC not reached) and *M. pachydermatis* (MBC = 6.3 µg/mL) (Sala et al. 2018). Notably, this study represents the first evaluation of NCP-3 against reference strain of *S. pseudintermedius*, for which a strong bactericidal effect was observed (MBC = 0.8 µg/mL).

Time–kill assays confirmed the rapid bactericidal activity of NCP-3, particularly against *S. aureus*, MRSA, *S. pseudintermedius*, and *M. pachydermatis*, achieving complete inhibition within 5 min. In contrast, complete inhibition required longer exposure times for Gram-negative bacteria, consistent with previous reports (Sala et al. 2018), who reported a rapid bactericidal activity of NCPs, with complete inhibition occurring within 10 min for Gram-positive bacteria and 60–120 min for Gram-negative species.

Numerous studies have reported the antimicrobial activity of various AMPs—derived from animals, plants, or bacteria—against multidrug-resistant organisms (MDROs), including vancomycin-resistant *Staphylococcus aureus*, carbapenem-resistant *Klebsiella pneumoniae*, and multidrug- or extensively drug-resistant *P. aeruginosa* (Jarosiewicz et al. 2020; Mangoni et al. 2008; Oliveira Júnior et al. 2025). This study also represents the first evaluation of NCP-3 against a large and heterogeneous collection of clinical isolates from dogs with otitis externa, most of which were classified as multidrug-resistant organisms (MDROs) (Magiorakos et al. 2012).

Among Enterobacteriaceae, resistance was frequently observed against aminoglycosides, β-lactams, penicillins combined with β-lactamase inhibitors, and quinolones. Nearly all staphylococcal isolates were resistant to oxacillin, confirming the presence of phenotypically methicillin-resistant strains. The high level of resistance observed among Pseudomonadaceae can be partially attributed to their intrinsic resistance to several antimicrobial classes commonly used in veterinary medicine; however, the detection of additional multidrug-resistance patterns is particularly concerning, as it further limits the already restricted therapeutic options available. Within the *M. pachydermatis* group, three clinical isolates showed resistance to ketoconazole, miconazole, or fluconazole, respectively (Spadini et al. 2025).

Overall, clinical isolates exhibited higher MBC values compared with reference strains. This was particularly evident for Enterobacteriaceae and *M. pachydermatis*, for which neither MBC₅₀ nor MBC₉₀ values could be determined, and for Pseudomonadaceae, for which only MBC₅₀ was achieved. In contrast, *S. pseudintermedius* clinical isolates showed susceptibility profiles comparable to reference strains. Our findings are consistent with previous study which reports similar susceptibility profiles between clinical and reference *Staphylococcus* spp. isolates tested with AMPs (Jarosiewicz et al. 2020). The concentration range tested (≤ 12.5 µg/mL) was selected to reflect clinically relevant levels and those achievable in topical formulations, enabling a translationally meaningful assessment of NCP-3 activity. However, this approach may have limited the determination of MBC₅₀ and MBC₉₀ values for less susceptible clinical isolates, particularly among Gram-negative bacteria and *M. pachydermatis*. In these cases, a substantial proportion of isolates exhibited MBC values exceeding the highest concentration tested, preventing reliable calculation of these parameters.

The time–kill assay further highlighted differences between microbial groups. While rapid and complete inhibition was consistently observed for *S. pseudintermedius*, other microorganisms showed variable responses. Enterobacteriaceae displayed the greatest heterogeneity, whereas Pseudomonadaceae and *M. pachydermatis* showed a more progressive but incomplete reduction in viability. Notably, full bactericidal activity was observed within 5 min for all *S. pseudintermedius* clinical isolates. Clinical isolates belonging to the Enterobacteriaceae family exhibited the greatest heterogeneity across all time points, and even after prolonged exposure, the mean inhibition did not exceed 75%, largely due to the presence of poorly susceptible clinical isolates. In contrast, lower variability was observed among Pseudomonadaceae and *M. pachydermatis* clinical isolates. In these groups, a progressive increase in antimicrobial activity over time was evident, reaching mean inhibition values of 98% and 90%, respectively, after 120 min of exposure, indicating a limited presence of poorly susceptible clinical isolates.

A marked variability in susceptibility was observed among clinical isolates, particularly within Enterobacteriaceae and *M. pachydermatis*. This variability likely reflects intrinsic and adaptive tolerance mechanisms rather than specific acquired resistance. Since AMPs act through electrostatic interactions with microbial surfaces followed by membrane disruption, variations in cell envelope composition can significantly influence susceptibility. Similar findings have been reported for other AMPs, such as temporins derived from *Rana temporaria*, which showed greater activity against Gram-positive than Gram-negative bacteria, particularly when tested under conditions mimicking physiological environments (Mangoni et al. 2008).

In Gram-negative field bacteria, the reduced activity observed against clinical isolates may be explained by multiple factors. The outer membrane represents a major permeability barrier that can limit peptide access to the cytoplasmic membrane. In addition, clinical isolates may exhibit modifications of lipid A, such as the incorporation of positively charged moieties (e.g., 4-amino-4-deoxy-L-arabinose or phosphoethanolamine), which reduce the net negative surface charge and consequently decrease electrostatic interactions with cationic peptides (Needham and Trent 2013). Furthermore, the production of extracellular polymers, outer membrane vesicles, and biofilm matrix components may contribute to peptide sequestration and reduce effective concentrations at the target site (Oliveira Júnior et al. 2025). The high heterogeneity observed within Enterobacteriaceae clinical isolates may reflect differences in the expression of these mechanisms, including variability in membrane permeability, efflux systems, and stress response pathways (Ebbensgaard et al. 2015). These features, which are not necessarily specific to AMPs, may confer a broader adaptive advantage under environmental and host-associated conditions.

A similar trend was observed for *M. pachydermatis*, for which clinical isolates showed reduced susceptibility compared with the reference strain. This may be related to its lipid-rich cell envelope, which can limit peptide penetration, as well as to biofilm formation and extracellular lipid material that may reduce AMP efficacy (Cannizzo et al. 2007; Čonková et al. 2022). Given the limited available data, these findings highlight the need for further investigation. To date, this study represents the first investigation on the activity of AMPs on *M. pachydermatis* from dogs. While NCP-3 showed promising activity against reference strains, neither MBC₅₀ nor MBC₉₀ values were achieved for field isolates at the tested concentrations. Nevertheless, in time–kill assays only after 120 min of exposure, a mean of 90% of *M. pachydermatis* strains inhibition was observed. A direct comparison with previous studies is challenging, as most investigations have focused on *Candida* species (*C. albicans*,* C. glabrata*,* C. auris*, and *C. tropicalis*), for which variable susceptibility to AMPs has been reported (Raman et al. 2015; Vélez et al. 2024). Further studies are therefore needed to better characterize the interaction between AMPs and *M. pachydermatis*.

Importantly, these findings should also be interpreted in the context of canine otitis externa, which is frequently a polymicrobial condition involving both Gram-negative bacteria, such as *Pseudomonas aeruginosa*, and yeasts including *M. pachydermatis*. In such environments, microbial interactions and biofilm formation may further impair antimicrobial efficacy and contribute to the variability observed in vitro. Therefore, the reduced susceptibility of clinical isolates to NCP-3 may reflect, at least in part, adaptation to the complex ecological conditions of the infected ear canal.

The higher susceptibility of reference strains compared with clinical isolates likely reflects their adaptation to controlled laboratory conditions, whereas clinical isolates are shaped by host-associated pressures that may select for increased tolerance to membrane-active compounds.

Further studies are needed to better characterize NCP-3 activity against Gram-negative bacteria and *M. pachydermatis*, including its potential antibiofilm effects.

Considering the previously reported low cytotoxicity of NCP-3, even at relatively high concentrations (up to 100 µg/mL) (Sala et al. 2018), its topical administration for the treatment of canine otitis externa appears promising. However, a limitation of the present study is the lack of direct cytotoxicity assessment on canine-derived cells, such as keratinocytes, which are particularly relevant for evaluating safety in this context.

Overall, the favorable safety profile and rapid bactericidal activity of NCP-3 support its potential as a topical therapeutic agent for canine otitis externa. However, higher concentrations and optimized formulations may be required to achieve effective activity against Gram-negative bacteria and yeasts *in vivo.* In addition, the complex microenvironment of the ear canal, including cerumen, host-derived components, and microbial biofilms, may significantly affect peptide activity by limiting diffusion and promoting peptide sequestration, potentially reducing effective local concentrations. This highlights the importance of optimized formulation strategies to enhance peptide stability, penetration, and retention time in vivo.

## Conclusions

In this study, we evaluated the antimicrobial activity of NCP-3 against a broad panel of bacterial and *M. pachydermatis* clinical isolates obtained from dogs with otitis externa, including multidrug-resistant strains. The results indicate that NCP-3 exerts marked antimicrobial activity, particularly against *S. pseudintermedius*, whereas a more heterogeneous and generally reduced activity was observed on Gram-negative and *M. pachydermatis* clinical strains.

Overall, these findings, obtained under in vitro conditions, suggest that NCP-3 is primarily active against Gram-positive bacteria, including multidrug-resistant *S. pseudintermedius*. However, its limited and variable efficacy against Gram-negative bacteria and *M. pachydermatis* may restrict its antimicrobial spectrum. Further studies are needed to assess its safety, efficacy, and potential clinical applicability in vivo.

## Supplementary Information

Below is the link to the electronic supplementary material.

Supplementary Material 1 (DOCX 110 KB)

## Data Availability

Data are available on request from the authors.
