# Supplementary material for: In vitro antimicrobial activity of Naja cardiotoxin peptide-3 against clinical isolates from canine otitis
Source: Vet Res Commun. 2026 May 23;50(4):343. doi: 10.1007/s11259-026-11284-3 (PMC13198450; doi:10.1007/s11259-026-11284-3)

***Veterinary Research Communication***

***In vitro* antimicrobial activity of *Naja* Cardiotoxin Peptide-3 against clinical isolates from canine otitis**

Costanza Spadini, Nicolò Mezzasalma*, Alessandro Cupola, Simone Taddei, Clotilde Silvia Cabassi.

University of Parma, Department of Veterinary Science, Via del Taglio 10, 43126, Parma, Italy

*Corresponding author: nicolo.mezzasalma@unipr.it, Department of Veterinary Science, Parma, Italy.

S-Table 1. Table S1. Identification of clinical isolates included in the study, organized by microbial group. For each isolate, strain number, species identification, and corresponding identification method are reported. Gram-negative bacteria (Pseudomonadaceae and Enterobacteriaceae) were identified using the API® system, and identification confidence is expressed as percentage values. *Staphylococcus pseudintermedius* isolates were identified by MALDI-TOF MS at an external reference laboratory. *Malassezia pachydermatis* isolates were identified by PCR amplification of the ITS-1 region. The table provides a comprehensive overview of the distribution of isolates across species and the methods used for their identification.

| Pseudomonadaceae | | Identification | Enterobacteriaceae | | | Identification |
| --- | --- | --- | --- | --- | --- | --- |
| N° | Strain | API^®^ | N° | Strain | | API^®^ |
| 1 | *Pseudomonas aeruginosa* | 98.9% | 6 | *Enterobacter cloacae* | | 97.7% |
| 2 | *Pseudomonas aeruginosa* | 99.9% | 7 | *Escherichia coli* | | 99.9% |
| 3 | *Pseudomonas aeruginosa* | 99.9% | 10 | *Escherichia coli* | | 99.9% |
| 4 | *Pseudomonas aeruginosa* | 98.9% | 20 | *Escherichia coli* | | 99.8% |
| 5 | *Pseudomonas aeruginosa* | 98.9% | 38 | *Proteus mirabilis* | | 99.9% |
| 8 | *Pseudomonas aeruginosa* | 99.5% | 55 | *Escherichia coli* | | 99.9% |
| 9 | *Pseudomonas aeruginosa* | 99.5% | 56 | *Escherichia coli* | | 99.8% |
| 11 | *Pseudomonas aeruginosa* | 98.9% | 57 | *Proteus mirabilis* | | 99.9% |
| 18 | *Pseudomonas aeruginosa* | 99.9% | 58 | *Proteus mirabilis* | | 99.9% |
| 19 | *Pseudomonas aeruginosa* | 98.9% | 59 | *Escherichia coli* | | 99.9% |
| 21 | *Pseudomonas aeruginosa* | 99.9% | 60 | *Escherichia coli* | | 99.9% |
| 24 | *Pseudomonas aeruginosa* | 99.9% | 61 | *Enterobacter cloacae* | | 98.9% |
| 25 | *Pseudomonas aeruginosa* | 99.5% | 62 | *Proteus mirabilis* | | 99.9% |
| 26 | *Burkholderia cepacia* | 99.6% | 63 | *Escherichia coli* | | 99.8% |
| 28 | *Burkholderia cepacia* | 99.5% | 67 | *Escherichia coli* | | 99.9% |
| 30 | *Pseudomonas aeruginosa* | 99.9% | *M. pachydermatis* | | | Identification |
| 32 | *Pseudomonas aeruginosa* | 99.5% | N° | | Strain | PCR |
| 36 | *Burkholderia cepacia* | 99.9% | 39 | | *Malassezia pachydermatis* | *ITS-1* |
| 37 | *Pseudomonas aeruginosa* | 98.9% | 40 | | *Malassezia pachydermatis* |  |
| 54 | *Pseudomonas aeruginosa* | 99.9% | 41 | | *Malassezia pachydermatis* |  |
| 65 | *Pseudomonas aeruginosa* | 98.9% | 42 | | *Malassezia pachydermatis* |  |
| *S. pseudintermedius* | | Identification | 43 | | *Malassezia pachydermatis* |  |
| N° | Strain | MALDI-TOF | 44 | | *Malassezia pachydermatis* |  |
| 13 | *Staphylococcus pseudintermedius* | External lab | 45 | | *Malassezia pachydermatis* |  |
| 14 | *Staphylococcus pseudintermedius* |  | 46 | | *Malassezia pachydermatis* |  |
| 15 | *Staphylococcus pseudintermedius* |  | 47 | | *Malassezia pachydermatis* |  |
| 16 | *Staphylococcus pseudintermedius* |  | 48 | | *Malassezia pachydermatis* |  |
| 17 | *Staphylococcus pseudintermedius* |  | 49 | | *Malassezia pachydermatis* |  |
| 22 | *Staphylococcus pseudintermedius* |  | 50 | | *Malassezia pachydermatis* |  |
| 23 | *Staphylococcus pseudintermedius* |  | 51 | | *Malassezia pachydermatis* |  |
| 27 | *Staphylococcus pseudintermedius* |  | 52 | | *Malassezia pachydermatis* |  |
| 29 | *Staphylococcus pseudintermedius* |  |  | | | |
| 31 | *Staphylococcus pseudintermedius* |  |  |  |  |  |
| 33 | *Staphylococcus pseudintermedius* |  |  |  |  |  |
| 34 | *Staphylococcus pseudintermedius* |  |  |  |  |  |
| 35 | *Staphylococcus pseudintermedius* |  |  |  |  |  |
| 53 | *Staphylococcus pseudintermedius* |  |  |  |  |  |
| 64 | *Staphylococcus pseudintermedius* |  |  |  |  |  |
| 66 | *Staphylococcus pseudintermedius* |  |  |  |  |  |

S-Table 2. Antimicrobial Susceptibility Testing (AST) of bacterial clinical isolates.

|  | Bacteria Strains | Kirby Bauer | | | | | | | | | | | | | | | MIC  µg/mL  PB |
| --- | --- | --- | --- | --- | --- | --- | --- | --- | --- | --- | --- | --- | --- | --- | --- | --- | --- |
|  |  | AUG | CXN | CFR | CVN | CIP | CD | DXT | ENR | FFC | GMN | MAR | OT | OXA | PRA | TS |  |
| *1* | *P. aeruginosa* | R | R | R | R | R | R | I | R | R | I | S | S | R | S | R | ≤0,5 |
| *2* | *P. aeruginosa* | R | R | R | R | R | R | I | R | R | R | R | I | R | R | R | ≤0,5 |
| *3* | *P. aeruginosa* | R | R | R | R | R | R | I | I | R | R | S | I | R | R | R | ≤0,5 |
| *4* | *P. aeruginosa* | R | R | R | R | R | R | S | I | R | I | S | S | R | I | R | ≤0,5 |
| *5* | *P. aeruginosa* | R | R | R | R | R | R | S | S | R | S | S | S | R | S | R | ≤0,5 |
| *6* | *E. cloacae* | R | R | R | R | I | R | S | S | I | S | S | S | R | S | S | ≤0,5 |
| *7* | *E. coli* | R | R | R | R | R | R | R | R | R | R | R | R | R | R | R | ≤0,5 |
| *8* | *P. aeruginosa* | R | R | R | R | R | R | R | R | R | R | I | R | R | R | R | ≤0,5 |
| *9* | *P. aeruginosa* | R | R | R | R | R | R | R | R | R | I | S | S | R | S | R | ≤0,5 |
| *10* | *E. coli* | S | R | S | R | R | R | R | I | I | S | I | I | R | S | R | ≤0,5 |
| *11* | *P. aeruginosa* | R | R | R | R | R | R | I | R | R | I | I | S | R | R | R | ≤0,5 |
| *12* | *S. pseudintermedius* | S | R | R | R | R | R | R | R | S | R | R | R | R | R | R | / |
| 13 | *S. pseudintermedius* | S | R | S | S | S | R | S | S | S | R | S | I | R | S | S | / |
| *14* | *S. pseudintermedius* | R | R | R | R | R | R | S | R | S | R | R | S | R | R | R | / |
| *15* | *S. pseudintermedius* | R | R | S | R | S | R | R | S | S | I | S | R | R | S | R | / |
| *16* | *S. pseudintermedius* | R | S | S | S | S | I | S | S | S | S | S | S | R | S | R | / |
| *17* | *S. pseudintermedius* | S | S | S | S | S | S | S | S | S | S | S | S | S | S | R | / |
| *18* | *P. aeruginosa* | R | R | R | R | R | R | R | R | R | R | R | S | R | R | R | 1 |
| *19* | *P. aeruginosa* | R | R | R | R | I | R | S | R | R | S | S | S | R | R | R | ≤0,5 |
| *20* | *E. coli* | R | S | S | S | I | R | I | S | R | R | S | R | R | S | R | ≤0,5 |
| *21* | *P. aeruginosa* | R | R | R | R | R | R | S | R | R | R | I | R | R | R | R | ≤0,5 |
| *22* | *S. pseudintermedius* | S | S | S | S | S | R | R | S | S | S | I | R | R | S | R | / |
| *23* | *S. pseudintermedius* | S | S | S | S | I | R | R | I | S | S | S | I | R | S | I | / |
| *24* | *P. aeruginosa* | R | R | R | R | R | R | I | R | R | R | R | R | R | R | R | ≤0,5 |
| *25* | *P.aeruginosa* | R | R | R | R | R | R | R | R | R | R | R | R | R | R | R | ≤0,5 |
| *26* | *B. cepacia* | R | R | R | R | R | R | R | R | R | R | R | R | R | R | R | ≤0,5 |
| *27* | *S. pseudintermedius* | R | R | S | I | S | S | I | I | S | S | I | R | R | S | R | / |
| *28* | *B. cepacia* | R | R | R | S | S | R | R | I | R | R | S | R | R | I | R | ≤0,5 |
| 29 | *S. pseudintermedius* | S | S | S | I | I | S | I | I | S | S | I | S | R | I | S | / |
| *30* | *P. aeruginosa* | R | R | R | R | R | R | R | R | R | R | R | R | R | R | R | ≤0,5 |
| 31 | *S. pseudintermedius* | R | R | R | R | R | R | R | R | I | R | I | R | R | R | R | / |
| 32 | *P. aeruginosa* | R | R | R | R | R | R | I | I | R | R | S | R | R | S | R | ≤0,5 |
| 33 | *S. pseudintermedius* | R | R | R | R | S | R | R | R | S | S | I | I | R | R | R | / |
| 34 | *S. pseudintermedius* | R | R | S | R | R | R | R | R | I | R | R | R | R | R | R | / |
| 35 | *S. pseudintermedius* | R | R | S | I | R | R | R | R | S | R | S | R | R | S | R | / |
| *36* | *B. cepacia* | R | R | R | R | R | R | R | R | R | R | R | R | R | R | R | ≤0,5 |
| *37* | *P. aeruginosa* | R | R | R | R | R | R | I | R | R | R | R | S | R | R | R | ≤0,5 |
| *38* | *P. mirabilis* | S | S | S | S | S | R | R | S | R | R | S | R | R | I | S | > 256 |
| 53 | *S. pseudintermedius* | R | R | R | R | R | R | R | R | R | R | R | R | R | I | R | / |
| *54* | *P. aeruginosa* | R | R | R | R | R | R | R | R | R | R | R | R | S | R | R | ≤0,5 |
| *55* | *E. coli* | R | R | R | R | R | R | S | R | S | R | R | R | S | R | S | ≤0,5 |
| *56* | *E. coli* | R | R | R | R | R | R | S | S | S | R | S | R | S | R | S | ≤0,5 |
| *57* | *P. mirabilis* | R | R | R | R | R | R | S | R | S | R | R | R | R | R | S | ≤0,5 |
| *58* | *P. mirabilis* | S | S | S | S | R | R | R | R | S | R | S | R | R | R | R | ≤0,5 |
| *59* | *E. coli* | R | R | R | R | R | R | R | R | S | R | R | R | R | R | R | ≤0,5 |
| *60* | *E. coli* | R | R | R | R | R | R | R | R | S | R | S | R | R | R | R | ≤0,5 |
| *61* | *E. cloacae* | R | R | R | R | R | R | R | R | S | R | R | R | R | R | R | ≤0,5 |
| *62* | *P. mirabilis* | R | R | R | R | R | R | R | R | R | R | R | R | R | R | R | > 256 |
| *63* | *E. coli* | R | S | R | S | I | R | S | R | R | R | R | R | S | R | R | ≤0,5 |
| *64* | *S. pseudintermedius* | S | S | S | R | S | I | S | S | S | S | S | S | R | S | S | / |
| *65* | *P. aeruginosa* | R | R | R | R | S | R | R | R | R | R | S | R | R | R | R | ≤0,5 |
| 66 | *S. pseudintermedius* | R | R | R | R | R | R | R | R | S | R | R | R | R | I | R | / |
| *67* | *E. coli* | R | R | R | R | R | R | R | S | S | R | S | R | S | R | S | ≤0,5 |

AUG= amoxicillin/clavulanic acid; CXN= cephalexin; CFR= Cefadroxil; CVN= Cefovecin; CIP= Ciprofloxacin, CD= clindamycin; DXT= doxycycline; ENR= enrofloxacin; FFC= florfenicol; GMN= gentamicin; MAR= marbofloxacin; OT= oxytetracycline; OXA= oxacillin; PRA= pradofloxacin and TS= trimethoprim + sulfamethoxazole

S-Table 3. Result of Minimum Inhibitory Concentration (MIC) of ketoconazole (KTZ), miconazole (MCZ) and fluconazole (FCZ) against clinical isolates of *Malassezia pachydermatis* (MP).


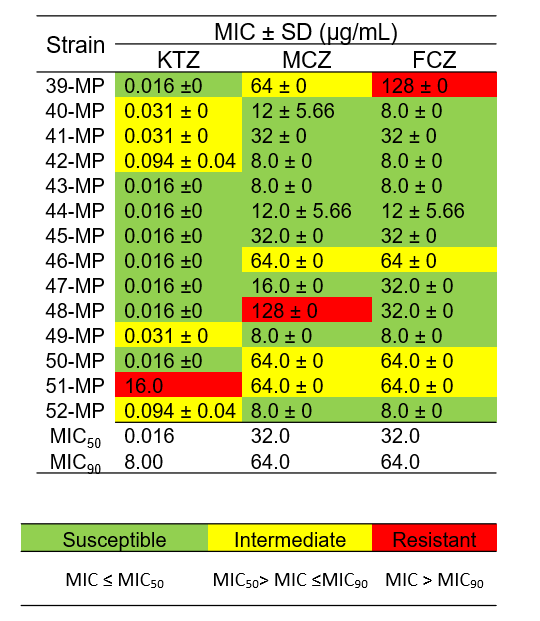

Supplement: Supplementary file 1 — Supplementary Material 1 (DOCX 110 KB) [file 11259_2026_11284_MOESM1_ESM.docx]
